# Seronegative Immune-Mediated Cerebellar Ataxia in Children: Autoimmune Encephalitis Spectrum Disorder or a Distinct Entity?

**DOI:** 10.3390/children12111513

**Published:** 2025-11-08

**Authors:** Gontika Maria, Tsimakidi Chrysanthi, Salamou Eudokia, Prattos Theofanis, Kallias Nikolaos, Kilidireas Constantinos, Tzartos John, Gkougka Dionysia

**Affiliations:** 1 Neurological Department, Penteli’s Children Hospital, 11528 Penteli, Greecesssgkgk@gmail.com (G.D.); 2 Medical School of National, Kapodistrian University of Athens, 11527 Athens, Greece; 3 First Department of Neurology, Medical School, National and Kapodistrian University of Athens, 11527 Athens, Greece; 4 Department of Neurology, Henry Dunant Hospital Center, 11526 Athens, Greece; 5 Second Department of Neurology, “Attikon” University Hospital, School of Medicine, National, Kapodistrian University of Athens, 12462 Athens, Greece; 6 Tzartos NeuroDiagnostics, 11523 Athens, Greece

**Keywords:** pediatric, seronegative, cerebellar ataxia, TIIF/IHC, biomarkers

## Abstract

Pediatric seronegative immune-mediated cerebellar ataxia (IMCA) remains a poorly defined and often under-recognized diagnosis, particularly in young children, where symptoms are frequently misattributed to self-limited post-infectious processes. We report the case of a 2.5-year-old girl who presented with acute-onset ataxia (mSARA score: 14). Cerebrospinal fluid analysis revealed pleocytosis and positive oligoclonal bands, while serial brain imaging and extensive autoantibody panels were unremarkable. However, indirect immunohistochemistry (TIIF/IHC) demonstrated a positive intracellular signal in cerebellar Purkinje cells, supporting the diagnosis of isolated seronegative IMCA. The patient showed sustained clinical improvement with prolonged corticosteroid therapy (mSARA score: 1). To date, only a few similar cases have been reported in the literature. It remains unclear whether these presentations fall within the spectrum of autoimmune encephalitis (AIE) or represent a distinct pediatric phenotype, potentially expanding the age range of primary autoimmune cerebellar ataxia previously described in adults. We recommend incorporating TIIF/IHC into the diagnostic workup of both isolated and combined pediatric cerebellar ataxia syndromes to support diagnosis and guide individualized treatment. Additionally, neurofilament light chain (NfL) and glial fibrillary acidic protein (GFAP) are emerging as promising biomarkers in this context and warrant further investigation.

## 1. Introduction

Acute ataxia in a previously healthy child is a rare presenting complaint, accounting for only 0.02% of all emergency department visits among children aged 1 to 18 years [1]. The differential diagnosis is broad, and although it includes potentially life-threatening conditions, post-infectious etiologies are the most common, responsible for 30–75% of cases [1].

Immune-mediated cerebellar ataxia (IMCA) associated with neuronal antibodies has only recently been recognized in children, typically occurring as part of the broader neurological manifestations of autoimmune encephalitis (AIE) syndromes, involving antibodies such as N-methyl-D-aspartate receptor (NMDAR), myelin oligodendrocyte glycoprotein (MOG), and glutamic acid decarboxylase 65 (GAD65) [2]. On the other hand, isolated IMCA is exceedingly rare, with only a few cases reported in the literature. These are frequently misdiagnosed, as many are tested negative for common autoantibodies in both serum and cerebrospinal fluid (CSF) [3].

Herein, we present the rare case of a 2.5-year-old girl with acute ataxia, detailing the comprehensive diagnostic workup that led to the diagnosis of isolated seronegative IMCA. We highlight the current uncertainty in classifying such cases, discuss the potential role of emerging serum biomarkers in monitoring therapeutic response, and provide a brief review of related cases from the literature.

## 2. Methods

The patient’s clinical data, imaging studies, serum, and CSF analysis were thoroughly reviewed. A modified version of the Scale for the Assessment and Rating of Ataxia (mSARA) was implemented for her evaluation [4].

Antibodies against MOG were assessed using an IgG1 live-cell-based assay, while antibodies against AQP4 and other cell-surface antigens were evaluated using fixed-cell-based assays. For antibodies against intracellular antigens, dot blot and immunohistochemistry in rat brain tissue were employed. CSF was further investigated for evidence of humoral autoimmunity. CSF was added to fixed rodent cerebellum and hippocampus frozen tissue, followed by incubation with a secondary FITC-labeled antihuman IgAGM antibody for visualization under a fluorescence microscope in order to search for any specific antibodies binding to brain tissue antigens.

Our report is in accordance with the CARE guidelines, and written informed consent was obtained from the patient’s caregivers. A brief review of related cases was conducted through targeted searches in PubMed and Google Scholar.

## 3. Case Report

A 2.5-year-old girl with an unremarkable medical history presented to the emergency department with an acute onset (less than 48 h) of right upper limb tremor and gait ataxia of cerebellar origin, characterized by a wide-based stance and an almost complete inability to stand without assistance (mSARA score: 14). The patient did not exhibit any prominent cognitive deficits or other extracerebellar manifestations. Upon admission, an extensive diagnostic workup was initiated (Table 1). Brain MRI was unremarkable, while lumbar puncture revealed elevated white blood cell count and weak type 2 oligoclonal bands (OCBs). During the first day of hospitalization, she was started on a five-day course of intravenous methylprednisolone (30 mg/kg), resulting in mild, gradual improvement. A comprehensive panel for autoantibodies in serum and CSF—including MOG, aquaporin-4, and antibodies against cell-surface and intracellular targets—returned negative results. At a three-month follow-up, she exhibited persistent deficits (mSARA score: 10) and transient symptom worsening during infections. Repeat investigations remained largely unremarkable (Table 1), prompting whole-genome sequencing (WGS) trio testing. Genetic testing revealed heterozygosity for a variant of uncertain significance (VUS) in the autosomal dominant gene *SLC1A3*, with the patient’s mother being homozygous for the same variant. Additionally, heterozygous variants were identified in the autosomal recessive genes *FA2H*, *SQSTM1*, *MRE11*, and *TDP1*, all of which were deemed clinically insignificant. Nevertheless, a brief trial of acetazolamide was undertaken to evaluate for possible episodic ataxia; however, it did not result in any clinical improvement.

**Figure 1:**
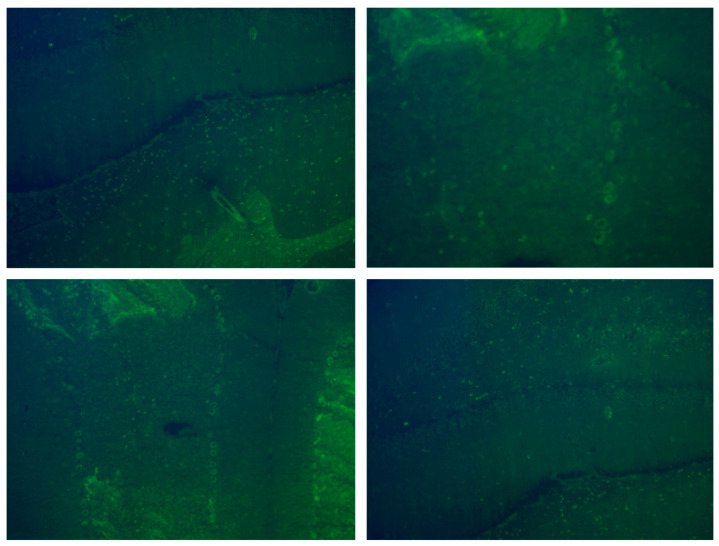
Tissue indirect immunofluorescence/immunohistochemistry (TIIF/IHC) of the patient’s cerebrospinal fluid on fixed rodent cerebellum, showing the positive intracellular signal of the Purkinje cells.

She subsequently received six monthly intravenous immunoglobulin (IVIG) infusions, resulting in moderate, time-limited improvement (mSARA score: 7). Midway through treatment, serum neurofilament light chain (NfL) and glial fibrillary acidic protein (GFAP) levels were measured and monitored longitudinally. Three months after the final IVIG infusion, tissue indirect immunofluorescence/immunohistochemistry (TIIF/IHC) on fixed rodent cerebellum and hippocampus revealed a positive intracellular signal in Purkinje cells (Figure 1). Whole-body MRI and urinary neuroblastoma markers were negative for occult malignancy. The patient was treated with a new three-day course of intravenous methylprednisolone (30 mg/kg), followed by six months of oral corticosteroid maintenance therapy (starting dose 2 mg/kg, with gradual tapering), leading to an excellent clinical response (mSARA score: 1) and a corresponding decline in serum biomarkers (Table 1). A year later, she remains relapse-free, with an mSARA score of 0 and stable biomarker levels.

## 4. Isolated Seronegative IMCA in Children: An Underdiagnosed Clinical Entity

Pediatric isolated seronegative IMCA is a rare, poorly defined, and often overlooked diagnosis. It includes cases of pure cerebellar ataxia without systemic or extracerebellar symptoms, presumed to be immune-mediated based on CSF and/or imaging findings, despite the absence of identifiable neuronal antibodies. Several challenges are usually encountered, including the need for early clinical suspicion of autoimmunity in the absence of multifocal CNS involvement, multiple negative antibody panels, and normal neuroimaging, particularly in a pediatric age group where ataxia is mostly attributed to benign, self-limiting post-infectious causes. In our case, despite our patient’s atypical clinical and imaging presentation, elevated CSF white blood cells, weakly positive oligoclonal bands, and partial response to short-course corticosteroids and monthly IVIG, as well as symptom fluctuation during infections, supported an underlying immune-mediated etiology. Subsequent TIIF/IHC revealed a positive intracellular signal in Purkinje cells, and a diagnosis of isolated seronegative IMCA was reached.

A review of the literature revealed one series of 36 Caucasian pediatric patients with acute cerebellitis, investigated for the presence of neuronal antibodies [3]. Of the total cohort, 72% were antibody-negative in both serum and CSF. Only four autoantibodies were identified—against MOG, GFAP, GlyR, and mGluR1 [3]. Clinical features were largely similar between antibody-positive and antibody-negative cases, although the latter had fewer MRI lesions and a lower rate of unilateral cerebellar involvement. Notably, 17% of patients had no cerebellar abnormalities on MRI, and 14% had completely normal scans, as in our case [3]. Severe complications, such as signs of obstructive hydrocephalus or MRI findings indicative of cerebellar herniation on MRI, occurred only in antibody-negative children, a finding potentially linked to their lower likelihood of receiving prolonged corticosteroid therapy. Researchers suggested that some antibody-negative patients may suffer from unidentified underlying causes that only partially respond to standard immunotherapies [3]. Only seven cases in the cohort were considered consistent with isolated seronegative IMCA; their key features are presented in Table 2.

In a second series involving 99 children of Asian descent presenting with acute ataxia of various etiologies [5], acute post-infectious cerebellar ataxia was the most common diagnosis, accounting for nearly 50% of cases. Only three children were identified as having primary autoimmune cerebellar ataxia (PACA), a term used by the authors to describe patients with suspected IMCA in whom no identifiable trigger or pathogenic neuronal antibodies have been found to date. No further details were provided about these specific cases [5].

## 5. Classifying Isolated Seronegative IMCA: A Variant of Pediatric AIE Spectrum or a Distinct Neurological Entity?

A key question that arises is whether these isolated cases should be viewed as part of the broader spectrum of pediatric AIE, possibly reflecting under-recognized or atypical phenotypes, or whether they represent a distinct neurological disorder.

While consensus criteria have been proposed for adult AIE [6], diagnosing AIE in children remains particularly challenging. A recent expert panel recommends considering autoimmunity in any previously healthy child with acute or subacute onset of new focal or diffuse neurological deficits, cognitive decline, developmental regression, movement abnormalities, psychiatric symptoms, and/or seizures [2]. Seizures—often treatment refractory—are the most common manifestation, typically accompanied by altered consciousness, disturbed sleep, and speech difficulties. Isolated presentations are considered less likely to raise suspicion for AIE, while abnormal movements, documented in 33%, are usually a supporting clinical feature, rather than an isolated manifestation, like in our case [2,7]. A completely normal brain MRI at onset is common; however, inflammatory changes or cerebral atrophy often emerge later in the disease course [2]. Comprehensive autoantibody panels in serum and CSF are standard in adult AIE evaluations [6], but in children, testing is typically limited to common targets such as NMDAR, MOG, GAD65, and gamma-aminobutyric acid receptor (GABA-R) [2,3,8]. Nevertheless, seronegative AIE is increasingly recognized in pediatric populations, accounting for over 70% of reported cases [8,9].

In adults, the term PACA has been introduced to describe a group of middle-aged patients (in their 50s) with suspected immune-mediated CA in which neither a trigger nor any pathogenic neuronal antibodies have been discovered yet [10,11]. Diagnostic criteria were introduced in 2020: (a) predominantly subacute or acute pure cerebellar syndrome; (b) MRI at presentation usually normal or may show primarily cerebellar vermian atrophy with (if available) reduced MR spectroscopy (NAA/Cr ratio) of the vermis; (c) at least two of the following: CSF pleocytosis and/or positive CSF-restricted IgG oligoclonal bands, history of other autoimmune disorders or family history of autoimmune disorders in first-degree relatives, or presence of antibodies that support autoimmunity but not yet shown to be either directly involved in ataxia pathogenesis or to be markers of ataxia with a known trigger; (d) exclusion of alternative causes made by an experienced neurologist or ataxia specialist (including other causes of immune ataxia such as PCD, GA, PIC and ones that are associated with well-characterized pathogenic antibodies) [11]. Our case meets the outlined criteria, raising the possibility that this entity also occurs in younger individuals. We believe it is important to clarify the potential incidence of this condition in the pediatric population, as affected patients may benefit from long-term immunomodulatory therapy, which could significantly improve their prognosis [12].

Notably, it has been argued that a substantial number of PACA cases still have a genetic defect that accounts for their ataxia that has not been identified [11]. In our patient, a comprehensive differential diagnosis was pursued, including screening for systemic autoimmune diseases, occult malignancies, and whole genome sequencing (Table 1). The genes in which variants were identified through genetic testing are associated with ataxia and cerebellar degeneration. However, the maternal homozygosity for the variant of uncertain significance (VUS) in *SLC1A3*, the lack of response to acetazolamide, along with the inheritance patterns and expression profiles of the other genes, do not support a correlation with the patient’s phenotype. Moreover, she achieved complete remission following treatment and continues to remain relapse-free—a clinical presentation that is inconsistent with the typical course of genetic ataxia.

Tissue indirect immunofluorescence/immunohistochemistry (TIIF/IHC) is a valuable tool and should be considered as the gold standard in the investigation of seronegative-IMCA, as unidentified by commercial panels antibodies may still be present [8,13,14]. Furthermore, differentiating between cell-surface and intracellular autoantibodies through TIIF/IHC can guide immunomodulatory treatment, promoting either IVIG or corticosteroids, as prompt and early initiation of treatment remains crucial for patients’ prognosis [15]. In our case, the presence of an intracellular rather than cell-membrane target prompted a thorough occult-malignancy workup, which was negative, and supported the use of prolonged corticosteroid therapy, leading to near-complete recovery more than a year after symptom onset [15,16].

## 6. The Role of Disease Biomarkers

In light of the growing body of research on disease biomarkers, neurofilament light chain (NfL) levels in CSF and serum have emerged as promising, non-invasive, and reproducible indicators of axonal damage across a wide range of neurological conditions [17]. Numerous studies have investigated their utility in predicting long-term outcomes and monitoring treatment response in both inflammatory and degenerative CNS disorders [17,18]. In a similar vein, glial fibrillary acidic protein (GFAP)—an intermediate filament protein primarily expressed by astrocytes in the CNS—has been widely employed as a biomarker of astroglial activation and CNS injury in various neurological diseases [19].

In our case, serum biomarkers served four key roles: (a) they indicated the presence of an active underlying process, prompting further investigation and diagnostic work-up; (b) their fluctuations in parallel with treatment and clinical status supported the inflammatory nature of the condition; (c) they demonstrated a strong correlation with disease remission to date; and (d) they have prompted continued clinical vigilance, as their levels have yet to fully normalize, suggesting the need for ongoing monitoring.

When comparing NfL and GFAP fluctuations in our patient, we observed that serum NfL levels varied in relation to treatment intervals but did not clearly reflect disease activity or remission status. This observation aligns with findings from other studies on AIE involving specifically antibodies against cell-surface antigens, where elevated NfL levels have been reported in both serum and CSF. However, no consistent associations have been established between NfL concentrations and CSF parameters or brain MRI findings [20,21], and a definitive correlation with clinical course remains unproven [20,22]. In contrast, GFAP—though rarely assessed in cases of AIE or IMCA—demonstrated a strong correlation with clinical severity. While GFAP levels remained relatively stable during IVIG therapy, they declined by approximately 40% following corticosteroid treatment and by 70% during remission, raising questions regarding the potential role of astrocyte-mediated inflammation in our case. Astrocytes play a dual role in the CNS [23]. They can contribute to pathology by releasing pro-inflammatory cytokines that recruit immune cells, activate microglia, and promote neurotoxicity. Conversely, they can also protect the brain by secreting anti-inflammatory cytokines and forming glial scars [23]. While nothing conclusive can be stated at this time, we believe that the role of astrocytes and GFAP warrants further investigation, as it may shed light on the underlying pathophysiological processes of the disease. Finally, the persistently elevated levels of both biomarkers warrant continued monitoring and further investigation. Evidence from studies in traumatic brain injury suggests that NfL levels can remain elevated for up to a year post-injury [24]. In our case, whether these sustained elevations reflect a chronic state, will decline over time or with additional immunotherapy, or may signal a risk of relapse remains uncertain. Clarifying these possibilities will require further research, ideally through studies involving larger patient cohorts.

## 7. Conclusions

Isolated seronegative IMCA remains under-recognized and unclassified in pediatric populations. Further research is needed to determine whether it falls within the AIE spectrum or represents a distinct entity, analogous to PACA in adults. We recommend incorporating TIIF/IHC into the diagnostic work-up of both isolated and combined cerebellar ataxia syndromes, as it can support diagnosis in seronegative cases and guide personalized treatment. Among emerging biomarkers, NfL and GFAP show potential for disease monitoring, underscoring the need for validation in larger cohorts.

## Figures and Tables

**Table 1:** Patient’s diagnostic workup.

Laboratory Test Time	CSF Analysis	OCBs	MOG/ AQP-4 Abs (Serum)	Abs Against Cell-Surface and Intracellular Antigens *	GFAP (Serum) pg/mL	NFL (Serum) pg/mL	WGS	Tissue-Based Immunohistochemistry	MRI Scan	Others
Baseline	WBC 30/μL, Protein 18 mg/dL, Glucose 50 mg/dL	Weak type 2	negative	negative					Brain: normal Cervical spine: normal	Systemic autoimmune panel: negative EEG: normal
3 months	WBC 0/μL, Glucose 60 mg/dL, Protein 57 mg/dL	Type 4	negative	negative						
6 months					325.0	9.5	VUS in SLC1A3–AD, mother homozygous **		Brain: normal Cervical spine: normal	
12 months		Type 1		negative	377	14.3		Hippocampus: negative Cerebellum: positive signal of Purkinje cells		Urine neuroblastoma and tumor blood markers: negative
18 months					222	9.2			Whole body: normal	
21 months					113	12.5				

CSF: cerebrospinal fluid; OCBs: oligoclonal bands; Abs: antibodies; MOG: myelin oligodendrocyte glycoprotein; AQP-4: aquaporin-4; GFAP: glial fibrillary acidic protein; NFL: neurofilament light chain protein; WGS: whole-genome sequencing; MRI: magnetic resonance imaging; WBC: white blood cell; EEG: electroencephalogram; VUS: variant of uncertain significance; SLC1A3: solute carrier family 1 member 3; AD: autosomal dominant. * NMDA-R, AMPA-R1/2, GABAB-R, LGI1, Dopamine-R2, DPPX, GluRδ2, IgLON5, mGluR1, mGluR5, CASPR2, GAD, Amphiphysin, CV2/CRMP5, PNAM2 (Ma2/Ta), Ri/ANNA-2, Yo/PCA-1, Hu/ANNA -1, Recoverin, SOX1, Zic4, Tr (DNER), ANNA-3, and AGNA (CSF and serum, cell-based assays and immunoblot). ** other findings: heterozygous in autosomal recessive genes FA2H, SQSTM1, MRE11, and TDP1.

**Table 2:** Reported cases of isolated seronegative IMCA in Caucasian children [3].

Age (Years)	Sex	Abs Status	Clinical Presentation	MRI Cerebellar Lesions	Additional MRI Lesions	CSF Findings	mRS (Onset)	mRS (Last f/u)
**6.2**	m	negative	ataxia, dysmetria, upper respiratory tract infection	unilat. lesions, vasogenic edema, gd (+)	none	pleocytosis, OCB (+)	4	2
**9.2**	m	negative	ataxia, dysmetria, headache, nausea, vomiting, fever	unilat. cortical lesion + vasogenic edema/swelling	none	pleocytosis, intrathecal IgG + IgM synthesis, OCB (+), enterovirus (PCR) *	3	1
**17.6**	m	negative	ataxia	bil. lesions, vasogenic edema/swelling	none	pleocytosis	2	2
**2.1**	m	negative	ataxia, dysmetria, dysarthria, respiratory tract infection, fever	bil. white matter/cortical lesions	none	pleocytosis, OCB (+), IgM *M. pneumoniae* abs	4	1
**7.4**	m	negative	ataxia, headache, vomiting	bil. lesions, vasogenic edema/swelling, cerebellar herniation	white matter, cortical/subcortical	pleocytosis	2	0
**2.5**	m	negative	ataxia, nausea, upper respiratory tract infection	none	none	pleocytosis	4	3
**5.1**	f	negative	ataxia, dysmetria, tremor, dysarthria, headache	none	none	pleocytosis, OCB (+)	4	3
**6.3**	f	negative	ataxia, vomiting, headache	unilat. lesions, vasogenic edema	none	none	3	1
**2.5**	f	negative	ataxia, tremor	none	none	pleocytosis, OCB (+)	3	0

IMCA: immune-mediated cerebellar ataxia; m: male; f: female; MRI: magnetic resonance imaging; CSF: cerebrospinal fluid; mRS: modified Rankin Score; f/u: follow-up; unilat.: unilateral; bil.: bilateral; abs: antibodies; OCB: oligoclonal band. * serum: Echovirus-IgM, Coxsackie A-IgM, Coxsackie B-IgM abs.

## Data Availability

The data presented in this study are available on request from the corresponding author.
